# IgG4-related nephritis and interstitial pulmonary disease complicated by invasive pulmonary fungal infection: a case report

**DOI:** 10.1186/s12882-020-02223-8

**Published:** 2021-01-11

**Authors:** Yili Xu, Guang Yang, Xueqiang Xu, Yaoyu Huang, Kang Liu, Tongfu Yu, Jun Qian, Xiufen Zhao, Jingfeng Zhu, Ningning Wang, Changying Xing

**Affiliations:** 1 Department of Nephrology, the First Affiliated Hospital with Nanjing Medical University, Jiangsu Province Hospital, Nanjing, China; 2 Department of Imaging, the First Affiliated Hospital with Nanjing Medical University, Jiangsu Province Hospital, Nanjing, China

**Keywords:** IgG4-Related nephritis, IgG4-Related lung disease, Corticosteroid, Invasive pulmonary fungal infection, Case report

## Abstract

**Background:**

IgG4-related kidney disease (IgG4-RKD) can affect multiple organs, which was first reported as a complication or extra-organ manifestation of autoimmune pancreatitis in 2004. It is characterized by abundant IgG4-positive plasma cells infiltration in tissues involved.

**Case presentation:**

A 69-year-old man presented with cough and renal dysfunction with medical history of hypertension and diabetes. Pathological findings revealed interstitial nephritis and he was initially diagnosed with IgG4-RKD. Prednisone helped the patient to get a remission of cough and an obvious decrease of IgG4 level. However, he developed invasive pulmonary fungal infection while steroid theatment. Anti-fungal therapy was initiated after lung puncture (around cavitary lung lesion). Hemodialysis had been conducted because of renal failure and he got rid of it 2 months later. Methylprednisolone was decreased to 8 mg/day for maintenance therapy. Anti-fungal infection continued for 4 months after discharge home. On the 4th month of follow-up, Chest CT revealed no progression of lung lesions.

**Conclusions:**

The corticosteroids are the first-line therapy of IgG4-RD and a rapid response helps to confirm the diagnosis. This case should inspire clinicians to identify IgG4-related lung disease and secondary pulmonary infection, pay attention to the complications during immunosuppressive therapy for primary disease control.

## Background

IgG4-related disease (IgG4-RD) is an inflammatory and fibrotic disease which was first described in the pancreas and was called autoimmune pancreatitis (AIP) in 2001 [1]. Its concept has been recognized worldwide since then that this systemic disease involved multiple organs or tissues characterized by elevated serum IgG4 level and IgG4 positive plasma cells infiltration in the affected tissues, leading to fibrosis eventually [2–6]. The prevalence of IgG4-RD in Japan was estimated as 0.28–1.08/100,000 people in 2012 [7].

IgG4-related tubulointerstitial nephritis (IgG4-TIN), is one of the frequent pathological changes of IgG4-related kidney disease (IgG4-RKD), accounting for about 15–25% of all IgG-RD [4, 8]. IgG4-related TIN shows a range of histologic appearances including (A) acute interstitial nephritis with minimal fibrosis; (B) a more cellular inflammatory pattern in the setting of expansile fibrosis; and (C) a very fibrotic, pauci-cellular pattern [7]. The diagnosis criteria of IgG4-RKD were proposed by the Japanese Society of Nephrology [9] and a work group of North America [7], respectively. IgG4-related kidney lesions were often associated with extrarenal disease, such as chronic sclerosing inflammation of the lacrimal gland, salivary gland [10] and lung [11].

Patients with IgG4-RKD have an increased risk of infection than general population. IgG4-TIN can be accompanied by eosinophilic lung disease [12] and pneumonia [13]. Here, we present a case of IgG4-RKD and lung interstitial lesions who developed invasive pulmonary fungal infection (IPFI) during treatment of glucocorticoid combined with immunosuppressive agents.

## Case presentation

**Fig. 1:**
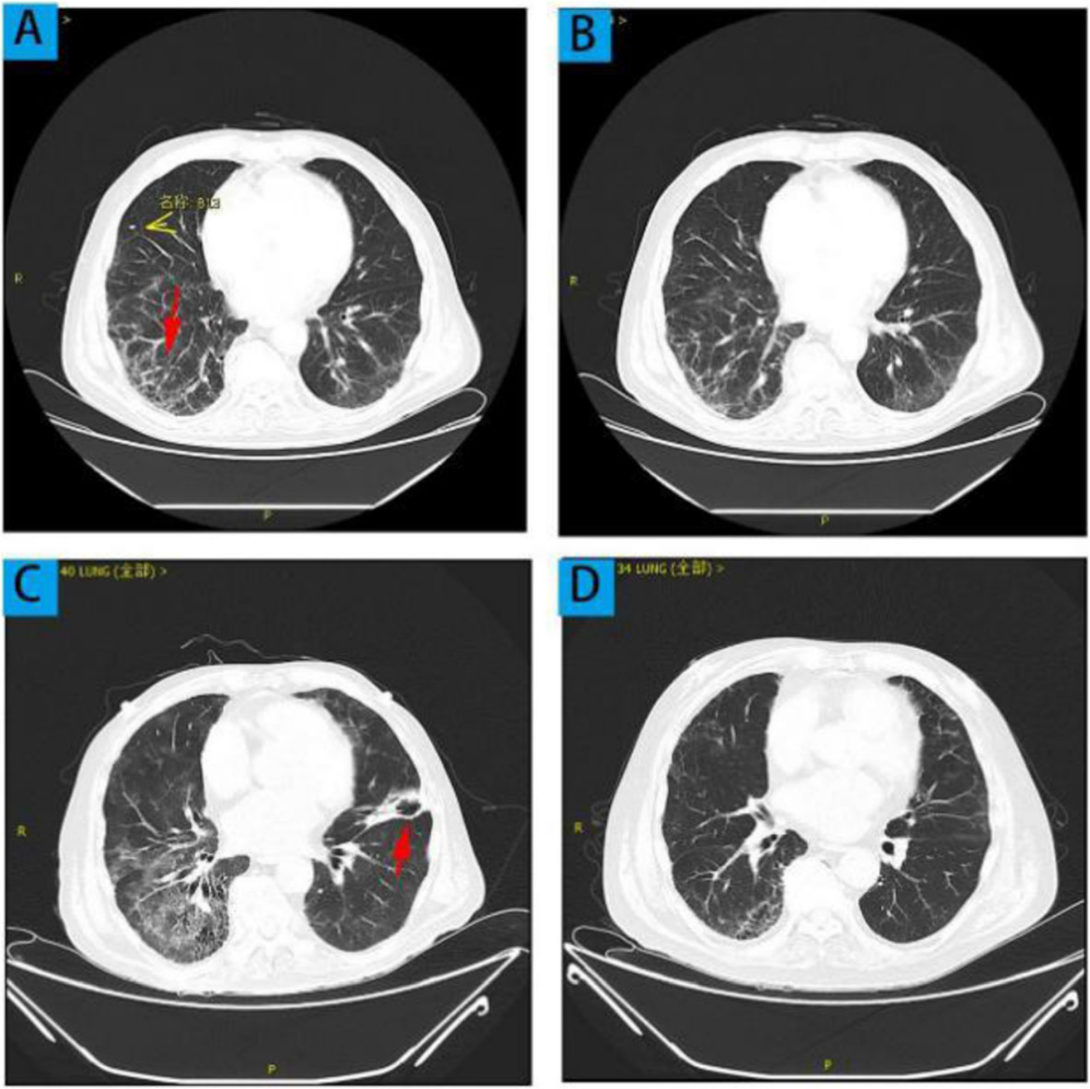
Chest CT showed interstitial inflammation and bilateral pleural effusion before therapy (**a**). After glucocorticoids admission, obvious absorption of interstitial inflammation and pleural effusion on both sides were found (**b**). Infection of both lungs with a left lower lobe cavity before anti-infection therapy (**c**). After anti-infection therapy, no increase boarder of lung lesions (**d**)

A 69-year-old male was admitted to the hospital in 2019 January 5^th^ because of gradually aggravated edema and cough. His medical history included hypertension, arrhythmia and diabetes. On 2019 May 8^th^, he had experienced cough and phlegm with temperature around 38 ~ 39 °C. Laboratory tests were presented in Table 1. ^18^F-FDG-PET/CT showed interstitial pneumonia in both lungs. There was also elevated uptake abnormality in the upper kidney observed. Cefperazone-Sulbactam, doxycycline hydrochloride, imipenem, and linezolid were given. Because of no improvement, he took oral prednisone 24 mg per day. The body temperature recovered to normal and lower limb edema was alleviated after one week. On 2019 June 13^th^ ,serum creatinine 157.7 umol/L (Fig. 1); and serum albumin, 24.9 g/L. Chest computed tomography (CT) scan showed that honeycomb-like changes considering interstitial inflammation and bilateral pleural effusion (Fig. 1A).

**Table 1:** Laboratory data performed before and after treatment

ITEMS	Beforetherapy	Steroidtherapyfor 1month		Steroid therapy for2 months	Steroid therapy for3 months	Steroid therapy for4 months
White blood cell count(*10^9/L)	15.4	21.11	8.24		11.66	13.28
Hemoglobin(g/L)	129	108	84		98	101
Platelet count(*10^9/L)	323	285	100		273	176
C-reaction protein (CRP)(mg/L)	90	58.1	32		79.8	18.9
Erythrocyte sedimentation rate(mm/H)	120	60	/		/	/
Serum nitrogen (mmol/L)	10.2	24.71	24.18		30.33	14.5
Serum creatinine (umol/L)	64.6	232.8	212.4		187.2	100.7
Serum albumin(g/L)	24.2	32	30		27.6	21.2
Urine RBC(/ul)	/	42.6	10.9			3
Urine protein creatine ratio(mg/g)		48.5				
24 h urine protein (g)	0.721	0.62	0.61		0.45	0.49
ANCA-MPO (RU/ml)		35.1	12.7		8.6	
ANCA-PR3 (RU/ml)		6.7	4.9		4.7	
CD4+ / CD8 + lymphocyte	0.81	/	/		/	1.9
ABG	Before therapy				Steroid therapy for 2 months	
PH	7.201				7.498	
PCO2(mmHg)	20				31.6	
PO2(mmHg)	117				49	
SpO2 (%)	98				88	
K(mmol/L)	5.4				4.1	
HCO3^-^(mmol/L)	7.8				24.5	
BE(mmol/L)	-20				1	

*ANCA-MPO *Myeloperoxidase-antineutrophil cytoplasmic antibody, ANCA-PR3 proteinase 3, *ABG *Arterial Blood Gas, / not available

He went to Nephrology Department on 2019 July 2^nd^ for further treatment. Laboratory results were presented in Table 1. Urine RBC 42.6/ul; Urine protein: creatine ratio (uPCR) 48.5 mg/g; Serum IgG4 level was elevated at 3.42 g/L (Normal range: 0.03–2.10 g/L). Anti-myeloperoxidase anti-neutrophil cytoplasmic antibody level was elevated at 35.1Ru/ml (Normal range:<20 Ru/ml). However, serum immunoglobulin A(IgA), IgG and IgM level were normal. Furthermore, the patient was negative for anti-double-stranded antibody, antinuclear antibody, anti-Sjogren’s syndrome A antibody, anti-Sjogren’s syndrome B antibody and anti-proteinase 3. Ultrasound displayed large-sized kidneys with uniform echo frequency and clear corticomedullary boundaries. Chest CT revealed obvious absorption of interstitial inflammation and pleural effusion on both sides, there were also multiple nodules in both lungs (Fig. 1b).

**Fig. 2:**
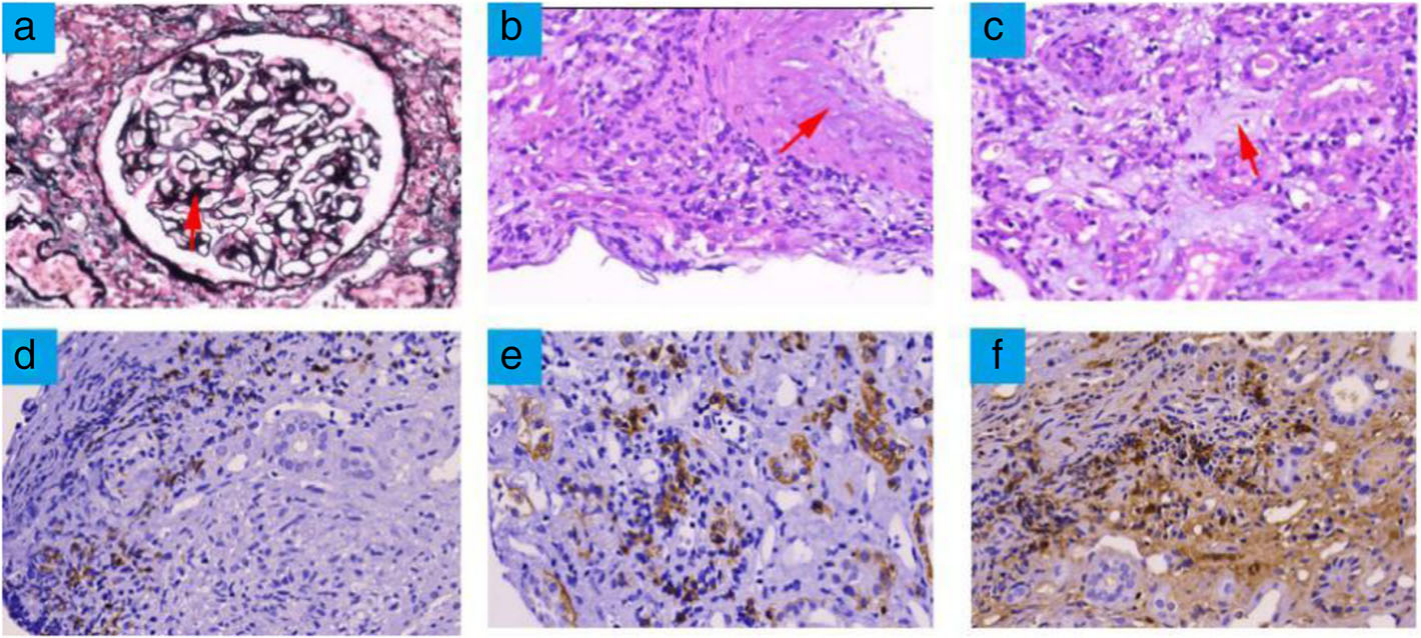
Renal histopathological result showed almost normal glomeruli, massively infiltrating cells, and abundant interstitial fibrosis under the light microscopy. **a** The glomerulus showed glomerular mesangial cells proliferation and diffuse and irregular thickening of basement membrane (HE staining, × 200). **b**, **c** A mass of plasma cells and fibrotic fibers (HE staining, × 400) can be observed in the interstitium. **d**, **e **Immunohistological analysis revealed mostly CD20-positive B cells (× 400) and CD138-positive plasma cells (× 400) in the interstitium. **f** Immunohistochemistry for IgG4 shows abundant positive plasma cells (coloured brown) (× 400)

**Fig. 3:**
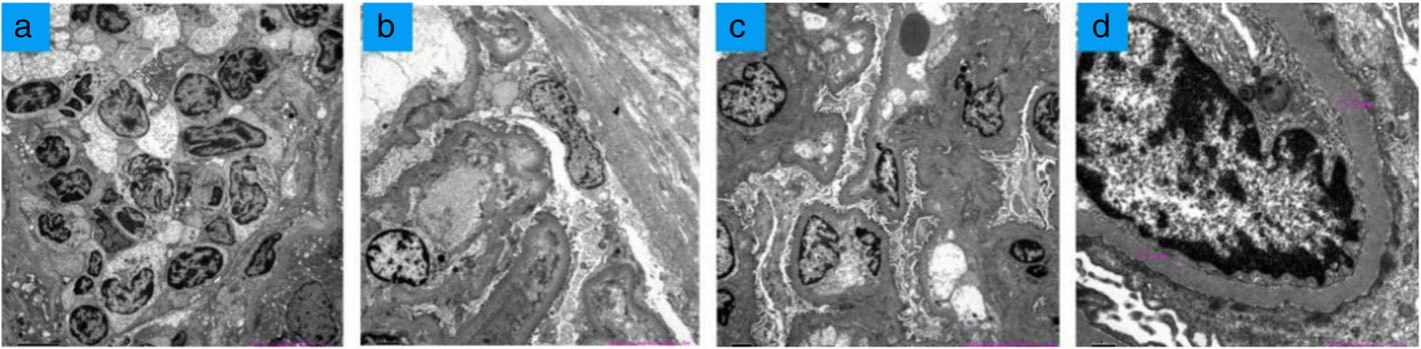
No electron-dense deposits are observed under electron microscopy. **a** Abundant plasma cells (× 2000) infiltrated in renal interstitium. **b** Detachment and partial atrophy of the microvilli of renal tubular epithelial cells as well as edema, infiltration of lymphocytes/monocytes, and fibrosis in renal interstitium. There are no electron-dense deposits in the glomeruli (× 2000). **c **Proliferation of glomerular mesangial cells and interstitial cells (× 2000). **d** Diffuse and irregular thickening of basement membrane (× 12,000)

Histopathology of the kidney biopsy shows proliferation of glomerular mesangial cells, diffuse and irregular thickening of basement membrane (Fig. 2a). The tubulointerstitium shows marked injury. Patchy foci fibrosis and inflammatory cells infiltration were prominent in the interstitium (Fig. 2b, c). Immunofluorescence staining shows that IgG, IgM, IgA, C1q, C3 and C4 were negative in the granular mesangial area. Immunohistological analysis revealed numerous CD20-positive B cells ((D), × 400) and dense infiltration of CD138-positive plasma cells ((E), × 400), with an IgG4^+^/IgG^+^ plasma cell ratio being > 40% ((F), × 400). Electron microscopy demonstrated that there were no electron-dense deposits in the glomeruli (Fig. 3).

**Fig. 4:**
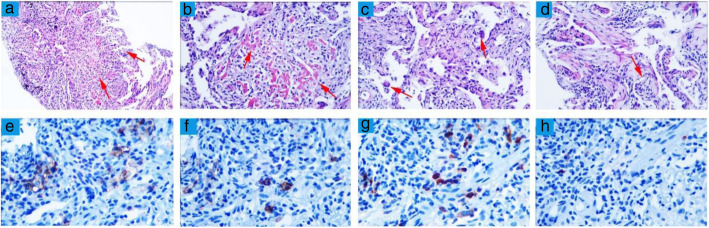
Histopathological findings of the pulmonary tissue (light microscopy: HE). **a** Pulmonary tissue shows fibrotic changes of the interstitium (× 100) in the lung. **b** Cell and tissue reactions and cellulosic exudation were observed in alveoli (× 200). **c**, **d** Giant cell, interstitial edema, collagen fiber hyperplasia and masson body were observed in the specimen (× 200). **e**, **f** Immunohistological analysis revealed CD38-positive plasmacyte (coloured brown) (E × 400) and CD138-positive plasmacyte (coloured brown) infiltration (F × 400). **g** IgG immunostaining shows IgG-positive plasmacyte (coloured brown) infiltration (× 400). **h** IgG4 immunostaining shows IgG4-positive plasmacyte (coloured brown) infiltration in the lung (× 400)

Based on these findings, he had been diagnosed as IgG4-related renal disease. Oral prednisone (40 mg/day) and cyclophosphamide (CTX, 0.4 g) were prescribed by intravenous infusion. The patient had been followed up every month after the treatment (Table 1). He presented with intermittent fever for more than 20 days and acute onset of left pleuritic chest pain with dry cough for 10 days. Negative results were found in aerobic or anaerobic blood culture. Chest CT showed recent infection of both lungs with left upper lung cavity (Fig. 1c). At June 28th he developed hemoptysis and type 1 respiratory failure (Table 2). The results of relevant tests are shown in Table 1. In order to differentiate between IgG4-RLD and IPFI, left upper lung puncture was conducted and showed that interstitial collagen fibrosis with acute and chronic inflammatory cell infiltration, focal fibrous necrosis and exudation, and small alveolar cell response. Fungal spores were also found in lung puncture specimen. Filamentous fungi can be seen in sputum culture. Immunohistochemistry test revealed that most plasma cells in the lung interstitium were positive for CD38 (+), CD138 (+), IgG (+) (Fig. 4). IgG positive plasma cells < 40%, IgG4 positive plasma cells < 10/HPF, which does not meet the pathological diagnostic criteria of IgG4 related diseases. The diagnosis of IPFI was definite. After then, the patient was initiated on voriconazole and caspofungin to anti-fungal infection, and prednisolone was decreased to 30 mg per day.

**Table 2:** Proposed diagnostic criteria for IgG4-RD according to ‘Comprehensive diagnostic criteria for IgG4-related disease’ [2, 14, 15]

Criterion	
Histology	(i)The specimen pathology shows the dense lymphoplasmacytic infiltrate, storiform fibrosis or obliterative phlebitis, the infiltration of IgG4-positive cells, and IgG cells more than IgG4 cells ratio of 40%;
Imaging	(ii)clinical/radiological examination showing characteristic diffuse or localized swelling or masses in single or multiple organs;
Serology	(iii) serum IgG4 concentration > 135 mg/dL; (iv)Inflammatory markers such as white blood cells count and C-reactive protein concentrations are not elevated, despite the degree of lesions, their spread on imaging analysis and massive cellular infiltration on pathological examination;
Other organ involvement	(v)Characteristic findings of IgG4-RD in other organs, including autoimmune pancreatitis, lung involvement, et al.
Treatment	(vi)response to steroids.

All 3 criteria (i + ii + iii) are needed for definite diagnosis of IgG4-RD

**Fig. 5:**
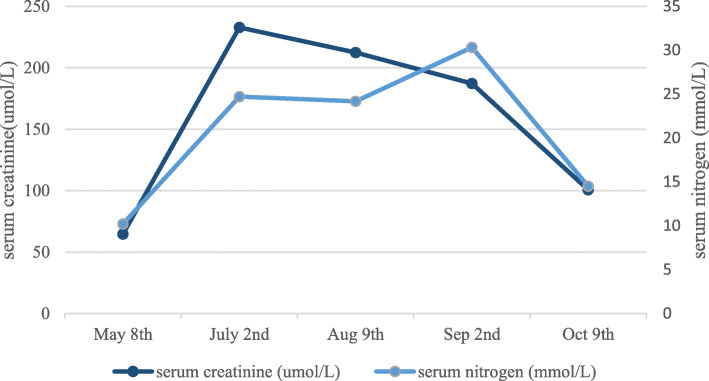
Dynamic changes of renal function before and after treatment in the patient with IgG4-RKD

With anti-infection and immunosuppressive treatment for 2 weeks, serum CRP and IgG4 level had been considerably decreased to 2.94 mg/L and 1.57 g/L, respectively. Voriconazole 200 mg bid and Methylprednisolone 30 mg/day were continued after discharge home. After treatment for one month, a repeat CT scan showed no progression of lung lesions (Fig. 1D). CD4^+^/CD8^+^ lymphocyte: 1.9. The patient had been followed up for 4 months. In the most recent follow-up examination, the serum creatinine level decreased to 101 umol/L and he got rid of hemodialysis (Fig. 5). He is currently undergoing tapered prednisolone treatment.

### Discussion and conclusions

This patient is characterized by serum IgG4 elevation, positive MPO-ANCA, which suggested the possibility of co-occurrence/concurrence of AAV and IgG4‐RD on his first visit from his serological presentation. Diagnosis of IgG4-RD requires particular pathological, serological and clinical features [2, 14, 15] (listed in Table 2). This patient presented with serum IgG4 elevation, hematuria, proteinuria, elevated uptake abnormality of the upper kidney observed in ^18^F-FDG-PET/CT, progressive kidney failure and interstitial lung disease. Histopathology of kidney biopsy showing typical lymphoplasmacytic infiltration and fibrosis enriched in IgG4-positive plasma cells, and infiltration of IgG4 ^+^ plasma cells with IgG4^+^/IgG^+^ plasma cells ratio greater than 40% and a total of ≥ 10 IgG4^ +^ plasma cells per high-power field (HPF) indicated the diagnosis of IgG4-RKD.

Given the elevated MPO-ANCA and CRP, AAV related nephritis was a possible differential diagnosis (Proposed diagnostic criteria for kidney involvement in AAV listed in Table 3 [16]). In this case, pathological findings did not show renal crescentic glomerulonephritis or vasculitis, thus, anti-neutrophil cytoplasmic antibody associated vasculitis (ANCA-AAV) was not firstly considered according to the Chapel Hill Consensus Conference nomenclature criteria for AAV [16]; However, Some ANCA positive patients may present with interstitial nephritis without severe glomerulopathy, and whether his initial interstitial lung disease was related to IgG4 or AAV or both was not determined. Some reports suggested a possible pathogenic effect of ANCA-IgG4 [17, 18]. Serum IgG4 increase and IgG4-positive cell infiltration in the organ can also be seen in AAV [19, 20]. Distinguishing between these diseases is essential for treatment planning [20], because IgG4-RD responds well to steroid therapy alone, while AAV often requires concomitant immunosuppressant use. His initial interstitial lung disease was improved (Fig. 1) with prednisone therapy alone for one month favored the diagnosis of IgG4-RLD. Kim et al. [21] described that a steroid trial was useful for differentiating and response to steroid therapy is recommended to be added to the diagnostic criteria. Proposed diagnostic criteria for IgG4-RD according to ‘Comprehensive diagnostic criteria for IgG4-related disease’ listed in Table 2. Therefore, we preferred his diagnosis of IgG4-RD.

**Table 3:** Proposed diagnostic criteria for kidney involvement in AAV [16]

clinical manifestations	a rapidly progressive GN with a decline in kidney function accompanied by sub–nephrotic-range proteinuria, microscopic hematuria, and hypertension over days to a few months
Serology	Anti-MPO antibody or anti-PR3 antibody positive
Pathological findings	pauci-immune focal necrotizing crescentic GN Rare patients with AAV have a prominent tubulointerstitial nephritis, which can be associated with vasculitis of the vasa recta

A diagnosis of AAV incorporates the integration of clinical features, ANCA serology, and tissue pathology as needed

Since kidney involvement was firstly reported in a patient with IgG4-RD in 2004 [22], many similar cases have been described [23–25]. A cross-sectional study reported in 2010 revealed all kidney lesions were associated with extrarenal disease among 114 patients with IgG4-related disease [26]. Several clinicopathologic studies reported IgG4-RD with both kidney and lung involvement [5, 14, 27–30]. In kidney its characteristic manifestation is TIN with multiple extrarenal tissue damage [14, 31], which is easily apparent with a chronic or rapid progressive renal function decline [23]. In lung, this may present as nodules with spiculated margins mimicking primary pulmonary malignancy [28, 32], multiple ground glass opacities (GGO) mimicking interstitial lung disease [14], alveolar interstitial type, and bronchovascular type [33].

However, he developed intermittent fever, acute onset of left pleuritic chest pain and an emerging lung lesion after steroid use for one month. IgG4-RLD has been classified into four categories based on CT. Our case was the GGO type. This also was the primary feature of IPFI [34] of which most common chest CT signs are nodules, consolidation and GGO. This patient had several high-risk factors of IPFI such as old age, long-term use of glucocorticoids, repeated hospitalization, etc. [35]. However,it is difficult to distinguish between IgG4-related lung disease (IgG4-RLD) and IPFI (Table 3). Lung puncture pathology is key standard. Thus, sputum culture and pathogenic examination were repeated and infiltration of IgG4-positive plasma cells was not found. The lung tissue specimen showed fungal spores which supported the diagnosis of IPFI. The differential diagnosis of IgG4-RLD and IPFI were listed in Table 4 [34, 36, 37].

**Table 4:** Differences between IgG4-RLD and IPFI [34, 36, 37]

Items	IgG4-RLD	IPFI
Clinical manifestations	multi-system injuries	dry cough and fever,no specific
Laboratory tests	Serum IgG4 elevation	CRP and (or) PCT elevation,G/GM positive
Imaging	nodules, multiple ground glass opacities (GGO),alveolar interstitial type, and bronchovascular type	nodules, consolidation and ground-glass opacity(GGO)
Pathology	Mainly IgG4 With plasma cell infiltration and often with interstitial damage	fungal spores with hyphae can be observed, pulmonary fibrosis and inflammatory cell infiltration
Treatment protocol	Systemic glucocorticoids	anti-infection

Systemic glucocorticoids are recommended as the first-line approach of renal injury in untreated IgG4-RD [31]. A moderate initial dose of oral prednisolone for induction is 0.6 mg/kg daily for 2–4 weeks. The maintenance dose of steroid therapy is given after remission as 2.5-5 mg daily over a period of 2–3 months [2]. However, treatment with exogenous glucocorticoids comes with a number of risks such as avascular necrosis, osteoporosis, glaucoma, cardiovascular disease, worse glucose tolerance and diabetes. The risk of infection is of utmost concern and is well-documented [36, 38]. A Japanese study including 459 AIP patients reported pneumonia occurred in 3 patients treated with steroid [13]. Optimizing the nutritional state of patients, reducing its dose, duration and number of immunosuppressants are recommended to help prevent infection. In the present case, we have to decrease the dosage of immunosuppressive drugs after then, nevertheless, IgG4-RLD were aggravated and renal failure developed during dosage decrease.

Since not every patient can be recover from renal disfunction, maintenance hemodialysis become necessary in patients with irreversible renal failure due to IgG4-RKD [4, 39]. And in this case, the patient experienced a short-time hemodialysis because of azotemia, which partly due to deteriorating renal function, steroids use or (and) infection. Improvements in pulmonary lesions and kidney function were observed after 4 months and were maintained with a dose of 8.0 mg/day prednisone. Thus, the dosage of steroid and immunosuppressant should be reduced for the therapy of the elderly patients with IgG4 related diseases. In addition, it has been reported that relapse of IgG4-related lesions, including kidney damage, occurred in 20% of treated patients with IgG4-RKD during maintenance treatment [39]. Thus, long-term follow-up for this patient are required and a well prognosis is expected.

**Fig. 6:**
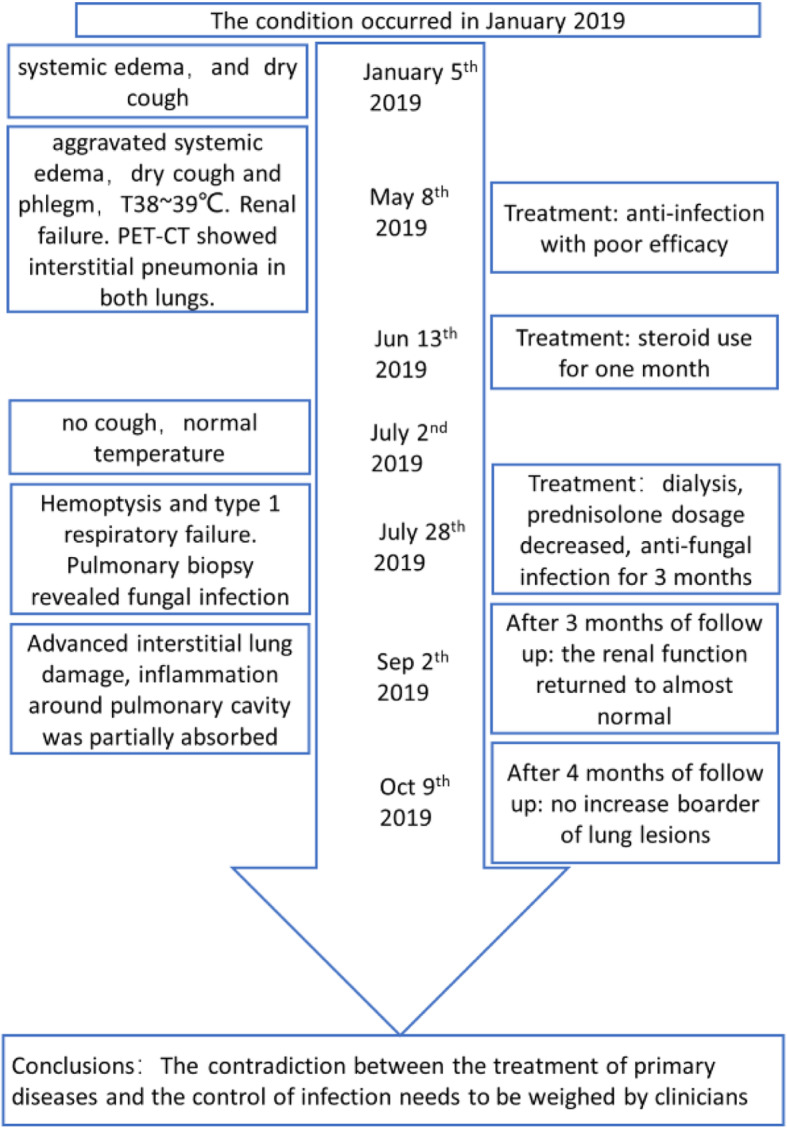
Flow diagram of the patient’s disease progression and treatment

Taken together, IgG4-RKD is an immune-mediated condition that can affect not only kidney but also several other organs, leading to a dense lymphoplasmacytic infiltration dominant in IgG4-positive plasma cells with fibrosis. This case should inspire clinicians to identify IgG4-related lung disease and secondary pulmonary infection, pay attention to the complications during immunosuppressive therapy for primary disease control (Fig. 6).

## Data Availability

The datasets used during the current study available from the corresponding author on reasonable request.

## Abbreviations

IgG4-RKD: IgG4-related kidney disease
AIP: Autoimmune pancreatitis
IgG4-TIN: IgG4-related tubulointerstitial nephritis
IPFI: Invasive pulmonary fungal infection
CT: Chest computed tomography
uPCR: Urine protein: creatine ratio
IgG: Serum immunoglobulin G
CTX: Cyclophosphamide
MPO-ANCA: Anti-myeloperoxidase anti-neutrophil cytoplasmic antibody
ANCA-AAV: Anti-neutrophil cytoplasmic antibody associated vasculitis
GGO: Multiple ground glass opacities
IgG4-RLD: IgG4-related lung disease
